# Successful treatment of severe, refractory polyomavirus disease with partially HLA-matched donor-derived BKPyV-specific T cells in a pediatric kidney recipient

**DOI:** 10.1007/s00467-026-07161-5

**Published:** 2026-01-19

**Authors:** Wibke Schumacher, Sophie Haumann, Lisa Eifler, Pablo Landgraf, André Oberthuer, Max Krause, Veronica Di Cristanziano, Britta Eiz-Vesper, Britta Maecker-Kohlhoff, Lutz T. Weber, Sandra Habbig

**Affiliations:** 1 Faculty of Medicine, Children’s and Adolescents’ Hospital, University Hospital of Cologne, University of Cologne, Cologne, Germany; 2 Department of Human Genetics, University Hospital of Cologne, Kerpener Str. 62, 50937 Cologne, Germany; 3 Institute of VirologyNational Reference Center for Papilloma- and Polyomaviruses, Faculty of Medicine and University Hospital Cologne, University of Cologne, Cologne, Germany; 4 Institute of Transfusion Medicine and Transplant Engineering, CELL Laboratory and T-Cell Registry, Hannover Medical School, Hanover, Germany; 5 Department of Pediatric Hematology and Oncology, Hannover Medical School, Hanover, Germany

**Keywords:** BK polyomavirus, BKV-associated nephropathy, Virus-specific T cells, JC polyomavirus

## Abstract

BK polyomavirus–associated nephropathy is a significant therapeutic challenge in kidney transplant recipients, often leading to allograft dysfunction. We report on a 12-year-old male kidney transplant recipient with severe, biopsy-proven BK polyomavirus–associated nephropathy and concurrent JC polyomavirus (JCPyV)–associated neurological symptoms. Due to failure of standard therapy, adoptive transfer of partially HLA-matched, BK polyomavirus–specific T cells from the kidney donor was administered as rescue therapy. The intervention induced a rapid decline in both BK polyomavirus (BKPyV) and JCPyV viral loads. This virological response was accompanied by the resolution of neurological symptoms and stabilization of allograft function. This case indicates that donor-derived BK polyomavirus–specific T cells represent a viable therapeutic modality for severe, refractory polyomavirus disease.

## Introduction

Reactivation of human polyomaviruses (hPyVs) due to impaired T cell immunity presents a significant risk to solid organ transplant (SOT) recipients. Specifically, BK polyomavirus (BKPyV) can cause BK polyomavirus–associated nephropathy (BKPyVAN), leading to allograft loss, while the genetically related human polyoma virus 2 (HPyV-2), formerly known as JC polyomavirus (JCPyV), can cause progressive multifocal leukoencephalopathy (PML). Adoptive cell therapy with virus-specific T cells (VSTs) is an emerging strategy to restore antiviral immunity in these scenarios. Due to significant protein homology, BKPyV-specific T cells (BKPyV-VSTs) exhibit cross-reactivity against JCPyV, offering a potential therapy for both infections. However, clinical experience with this approach for concurrent BKPyVAN and central nervous system JCPyV-disease is limited. We here report the successful use of donor-derived BKPyV-VSTs in a pediatric kidney transplant recipient suffering from BKPyVAN and JCPyV infection.

## Case report

The patient and his mother gave written informed consent to this case report. The patient presented with congenital nephrotic syndrome due to a homozygous mutation in the *MAGI2* gene. Hemodialysis was initiated at 10 years and continued until the patient received a living-donor kidney transplantation from his mother at the age of 12 years. Due to ABO incompatibility, he received rituximab and immunoadsorption prior to transplantation. Post-transplant immunosuppression (IS) included tacrolimus, mycophenolate mofetil, and prednisone. BKPyV and JCPyV viremia were first detected 3 months after transplantation, followed by a decline in allograft function at 10 months post-transplant. Kidney biopsy revealed severe BKPyVAN with 90% tubuloatrophy but no signs of transplant rejection. Reduction of tacrolimus, as recommended by recent guidelines [1], which was necessary to limit further progression of viral nephritis and encephalitis, was not effective in the reduction of viral load, neither was a complete cessation of calcineurin inhibitor and mycophenolate mofetil treatment with a switch to an everolimus-based regimen due to the potential antiviral effects of mTOR inhibitors [2]. The patient showed progressive signs of neurological disease, including epileptic seizures, tremor, cephalgia, visual impairment, and EEG abnormalities. Analysis of the cerebrospinal fluid revealed a normal cell count but elevated BKPyV and high JCPyV viral loads. While circulating JCPyV-specific antibodies were detectable in the serum, they were initially absent in the cerebrospinal fluid and became detectable 2 months after the first clinical presentation. Both a PCR panel analysis for other neurotropic viruses and a culture from cerebrospinal fluid resulted negative. According to recent literature, this condition might be considered PML even without the typical cerebral MRI findings [3]. EEG demonstrated increased cortical excitability without a clear ictogenic focus, supporting the diagnosis of a structural epileptic syndrome and aligning with the absence of an identifiable lesion on brain MRI. As reduction of immunosuppressive therapy and IVIG proved ineffective, no further treatment was available. When the patient’s condition deteriorated, we decided to offer treatment with partially HLA-matched BKPyV-VSTs. This approach is reserved for cases where the immune response is inadequate and when conservative options have failed. We decided to use T cells of the mother (kidney donor) with the lowest possible risk of rejection and with partial HLA matching. 

Following lymphocyte apheresis, manufacturing of clinical-grade BKPyV-specific CD4+ and CD8+ T cells was performed on a CliniMACS Prodigy device using MACS GMP Peptivators VP1 and LT in combination and the IFN-γ Cytokine Capture System (Miltenyi Biotec) with a purity of over 35% CD + IFN-γ-positive cells. A fresh product and eight cryopreserved products were prepared. Initially, 1 × 10^4^ CD3+ cells per kilogram (kg) bodyweight (bw) were administered by intravenous infusion following standard premedication. The patient was closely monitored for 48 h.

**Fig. 1:**
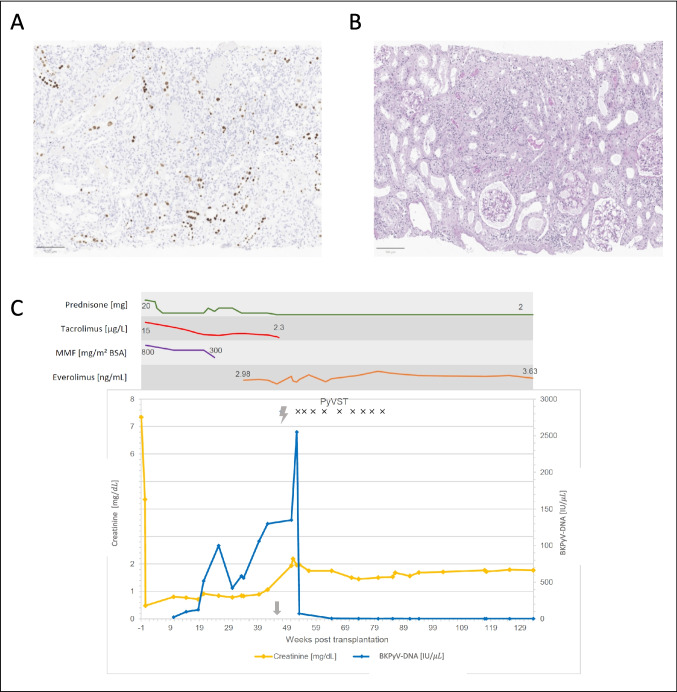
**A** Kidney biopsy in our patient: Immunostaining of SV40 with typical inclusion bodies representing polyomavirus nephritis. **B** PAS staining with tubuloatrophy and fibrotic areas. **C** Treatment and viral load in the presented patient showing an increase in BKV viral load and a concomitant rise in serum creatinine. Kidney biopsy was confirmed at around week 45 post-transplant (arrow). Significant reduction of viral load and creatinine after administration of BKPyV-CTL. Responding to the onset of neurological symptoms (lightning symbol), nine infusions of PyVST were administered (x). Lumbar punctures were performed at weeks 46, 51, 64, and 102. Yellow: creatinine [mg/dL]; blue: BKPyV-DNA [IU/µL] in serum

The patient received nine infusions at 4-week intervals. The individual dose was selected in consultation with experienced VST specialists. The recommended dose is 2.5 × 10^4^ CD + T cells/kg bw per infusion, which has been proven effective and safe in other viral complications such as PML [4]. Due to the excellent tolerability of the treatment, we decided to transfuse each of the cryopreserved aliquots containing 1.33 × 10^6^ cells (approximately 3.9 × 10^4^ CD3+ T cells cells/kg bw). Virological testing revealed a rapid reduction in BKPyV load (as shown in Fig. 1). BKPyV and JCPyV were cleared from cerebrospinal fluid. The treatment was well tolerated, with no severe side effects or immunological complications such as IRIS. Allograft function stabilized, and neurological symptoms completely resolved.

## Discussion

BKPyVAN poses a major challenge after kidney transplantation, potentially resulting in allograft loss. Standard post-transplant protocols include surveillance for BKPyV reactivation in plasma and urine. Presumptive BKPyVAN is diagnosed upon detecting a rise in viremia accompanied by deteriorating allograft function. Treatment is based on reduction of immunosuppressive therapy and/or switching to an mTOR inhibitor–based therapeutic regimen. Adjuvant therapies like intravenous immunoglobulins may be used.

Reactivation of JCPyV can manifest as PML—a potentially fatal demyelinating disease. Previous approaches including treatment with mirtazapine [5] or immune checkpoint inhibitors [6] have been of limited efficacy and are not eligible for pediatric patients under IS.

Adoptive cell therapy using VSTs is reserved for patients with severe, refractory, or drug-resistant viral infections, particularly when endogenous antiviral immunity is impaired. These antigen-specific T cells can be donor-derived or obtained from partially HLA-matched third-party donors [7] and are administered intravenously. Repeat dosing is guided by clinical response, virological kinetics, and tolerability. Reported adverse events include cytokine release syndrome [8] and graft-versus-host disease.

Safety and potential efficacy of treatment with directly isolated allogeneic VSTs in PML was described before. Möhn et al. demonstrated clinical benefit of BKPyV-VSTs in 22/28 patients with JCPyV-associated PML [9]. All infusions were well tolerated, and no serious treatment-related adverse events were observed within this study.

We here faced a case of concurrent active BKPyVAN plus progressive neurological symptoms with proof of JCPyV in the cerebrospinal fluid. We therefore decided to offer adoptive cell therapy with VSTs. We hypothesized that this approach might be effective in treating both BKPyVAN and JCPyV infection as evidence demonstrated the cross-reactivity and efficacy of BKPyV-VSTs against both BKPyV and JCPyV [10]. Further studies are needed to evaluate the risks and benefits of this approach in isolated BKPyVAN.

In the current case, the mother as a former kidney donor and with functional proof of BKPyV-VSTs in the peripheral blood was considered an ideal donor and agreed to the procedure. In cases where a family donor is not available, a registry (e.g., www.allocell.org) can be used to screen for a partially HLA-matched third-party donor. While the rejection risk for SOT recipients after third-party donation of VSTs cannot be determined with current knowledge, it must nevertheless be considered.

This adolescent patient did not exhibit endogenous BKPyV- or JCPyV-specific T cells in peripheral blood at any point prior to adoptive T cell therapy. An effect on viral load could be demonstrated after the first infusion of VSTs. In the cerebrospinal fluid, JCPyV viral load was undetectable and cerebrospinal fluid clearance occurred prior to the fourth infusion.

Following adoptive transfer, BKPyV- and JCPyV-specific T cells similarly remained undetectable during subsequent follow-up assessments. The patient undergoes monitoring every 3 months. Neurological symptoms have been in remission, and the graft function remains stable at a reduced level, reflecting the viral damage of tubuloatrophy. This case adds to the growing evidence that BKPyV-VSTs are a safe and successful rescue therapy for concurrent BKPyVAN and JCPyV neurological disease when conventional management fails.

A significant proportion of pediatric kidney transplantations involve living donation from a relative. Consequently, a unique therapeutic opportunity exists in this population, as the organ donor frequently represents an ideal, readily available source for generating BKPyV-VSTs. Prospective studies are needed to validate the safety, establish standardized protocols, and confirm the long-term efficacy of this potentially allograft-saving treatment.

## Summary

### What is new?

- Treatment with donor-derived BKPyV-VSTs proved safe and effective in severe, refractory polyomavirus disease in a pediatric kidney transplant recipient.

## Data Availability

This manuscript describes routinely collected clinical data, and no additional datasets or statistical analyses were generated. The underlying clinical information is not publicly available due to patient confidentiality but is available from the corresponding author on reasonable request.

## Abbreviations

BKPyVAN: BK polyomavirus–associated nephritis
BKPyV: BK polyomavirus
BKPyV-VSTs: BKPyV virus–specific T cells
bw: Bodyweight
HPyV-2: Human polyomavirus 2 (former name JCV)
hPyV: Human polyomavirus
IS: Immunosuppression
JCPyV: JC polyomavirus
kg: Kilogram
PML: Progressive multifocal leukoencephalopathy
SOT: Solid organ transplant
VSTs: Virus-specific T cells
